# Impact of COVID-19 on Pakistani dentists: a nationwide cross sectional study

**DOI:** 10.1186/s12903-021-01413-6

**Published:** 2021-02-10

**Authors:** Ramsha Kamran, Kiran Saba, Saima Azam

**Affiliations:** Operative Dentistry Department, Islamabad Medical and Dental College, Main Murree Road, Bharakahu, Islamabad, Pakistan

**Keywords:** Healthcare, Pakistan, Workplace, Public health, Infection control, Afraid

## Abstract

**Background:**

The COVID-19 outbreak which developed into a public health crisis has raised concerns regarding infection control among health care workers particularly dentists all over the world. The aim of this survey was to assess awareness, fear and compliance with practice modification according to CDC guidelines during COVID-19 pandemic among Pakistani dentists.

**Methods:**

A cross-sectional study was conducted using an online survey questionnaire. The questionnaire was designed on Google Forms and was distributed among all seven regions of Pakistan through social media and WhatsApp after carrying out the reliability analysis. Statistical analysis was performed using SPSS 20.0. Question wise analysis using frequencies and percentages was done. Pearson correlation and Kruskal Wallis test was applied to check association of awareness level with qualification and workplace setting.

**Results:**

A total of 313 dentists participated and submitted the form online from all regions of Pakistan. The response rate was quite satisfactory as Pakistan was under an official lockdown and most of the hospitals/clinics were either closed or operating with minimum staff. Most of the dentists were well aware of the CDC guidelines. However, 75% of the dentists were afraid of getting infected and 88% of them were anxious while providing treatment. Sixty-eight percent of them were avoiding aerosol generating procedures and only 28% were using rubber dam isolation. Regional analysis was also done and areas of poor compliance were identified. Only 38.5% dentists in Balochistan were using N95 masks and none of the dentists (0%) were using rubber dam isolation. A large number of dentists (80.9%) were afraid and wanted to close their dental practice in Khyber Pakhtunkhwa. Furthermore, a positive correlation was seen between the designation and awareness level (p = 0.01). Similarly, significant correlation (p = 0.03) was seen between qualification and workplace setting.

**Conclusion:**

The results of the study can help devise strategies to ensure adherence with infection control guidelines in regions with poor compliance. Initiation of awareness programs to help overcome fear and train the faculty and staff in the targeted areas would greatly contribute towards reducing the spread of infection and thus lowering the healthcare burden in a third world country like Pakistan.

## Background

The outbreak of coronavirus disease (COVID-19) was observed for the first time in December 2019 [1] and is believed to have originated in a seafood market in Wuhan City, China [2]. In January 2020, the World Health Organization (WHO) declared COVID-19 a public health emergency of international concern [3, 4] and within a few months the General Manager of WHO declared COVID-19, a pandemic which has spread all over the world [5]. COVID-19 pandemic is much more than a global health crisis of our time and the biggest challenge faced by us since World War II, according to United Nations Development Programme (UNDP) [6].

Coronavirus is thought to have a zoonotic origin [7] and structurally belongs to a single stranded RNA family with a size of ~ 350 kilo base-pair (kbp) [8, 9].The virus has the ability to cause severe acute respiratory tract infection and is highly contagious, spreading through saliva, hands, nasal droplets and less commonly through surface contact [10, 11].

Due to its rapid spread, coronavirus disease has caused wide spread public health concerns as entire international community is in its grip now [7]. A large number of health care workers have reported acquiring the disease while working with infected individuals. According to a note on worker exposure risk to COVID-19, published by Occupational Safety and Health Administration (OSHA), dental professionals fall into very high-risk exposure category for nosocomial infection and can become carriers of the disease due to their routinely aerosol generating procedures [12]. Similarly, an article published by the New York Times on March 2020 entitled “The worker who face the greatest coronavirus risk”, described that dentists are the most exposed workers, much more than nurses and general physicians [13]. Dentists therefore should take measures for prevention and entertain a high level of awareness to reduce risk of contagion from COVID-19 [14] and appropriate mitigation of further spread.

In face of current pandemic, anxiety is a normal and predictable response, but severe anxiety can lead to a state of panic leading to irrational behaviour especially in high risk profession like dentists [2]. Although Centers for Disease Control and Prevention (CDC), the American Dental Association (ADA) and WHO have published preventive guidelines to control the spread of COVID-19 [15], majority of dentists are still scared and unwilling to treat patients in this situation. In fact a lot of dentists might not even be aware of these guidelines [2].

In developing countries like Pakistan, it is still a debatable topic whether infection control protocols are religiously followed in dental clinics and OPDs as one of the prime factors influencing it, is finance or resources, reported by Dental News Pakistan on May 2020 [16]. This can be confirmed by a few previous studies showing an overall poor compliance and knowledge on use of personal protective equipments (PPEs) among dental students of a teaching hospital in Rawalpindi, Pakistan [17]. Similar results were seen in Karachi, Pakistan where only 20% of the respondents were compliant unlike in other countries like Kuwait, New Zealand, Saudi Arabia and Canada where knowledge and compliance was far better (84%) [18]. Therefore, lack of formal training/knowledge in infection control and limited resources is thought to be the reason of not adhering to cross infection control guidelines [19]. However, such negligence can not be afforded at any cost during this current pandemic as it can have catastrophic results leading to severe economic and healthcare burden in a third world country like Pakistan.

As there is very limited data available on a developing country like Pakistan during COVID-19 pandemic the purpose of this study is to assess the awareness, fear and compliance with the work practice modification among Pakistani dentists.

## Methods

A cross sectional survey was conducted using an online survey questionnaire from 16 to 20th June 2020. Ethical approval was obtained from Institute’s Research & Ethics Committee.

The questionnaire was designed on Google Forms and reliability analysis was undertaken by carrying out a pilot survey initially. The intra-class correlation showed a strong relation of 0.71. In order to achieve maximum possible outreach in all regions of Pakistan, all central locations of Pakistani practicing dentists were identified on social media and WhatsApp. After identification, the questionnaire was posted on the Pakistan Dental Forum (largest representation of Pakistani dentists on social media), facebook pages of renowned dental colleges across Pakistan and on WhatsApp groups representing the Pakistani Dental Community.

A structured questionnaire was prepared to assess the fear and compliance of Pakistani dentists with work practice modification during the COVID-19 pandemic (Additional file 1: COVID-19 Questionnaire). The questionnaire comprised of 32 closed ended questions of which 6 were about the dentists’ demographic information and 8 regarding fear and anxiety assessment during the COVID-19 outbreak. The questions regarding fear and anxiety were adapted from the questionnaire used in study conducted by Ahmed et al. in thirty different countries across the world [2]. Rest of the questions were related to the knowledge and practice modification of dentists in accordance with the updated CDC and ADA practice guidelines.

Data analysis was carried out using Statistical Package for the Social Sciences version 20.0 (SPSS 20.0). Frequencies and percentages were calculated for qualitative variables. Question wise analysis in terms of percentages for each response was done. Association of awareness level with designation/qualification and workplace setting was evaluated using Pearson correlation and Kruskal–wallis H test respectively.

## Results

A total of 313 practicing dentists participated and submitted the questionnaire from seven different regions of Pakistan. The response was quite satisfactory as the questionnaire was only filled by dentists who were practicing during the lockdown period and most of the hospitals/clinic were either closed or operating with minimum staff. The questionnaire comprised of 32 questions in three sections. All questions were mandatory.

**Fig. 1:**
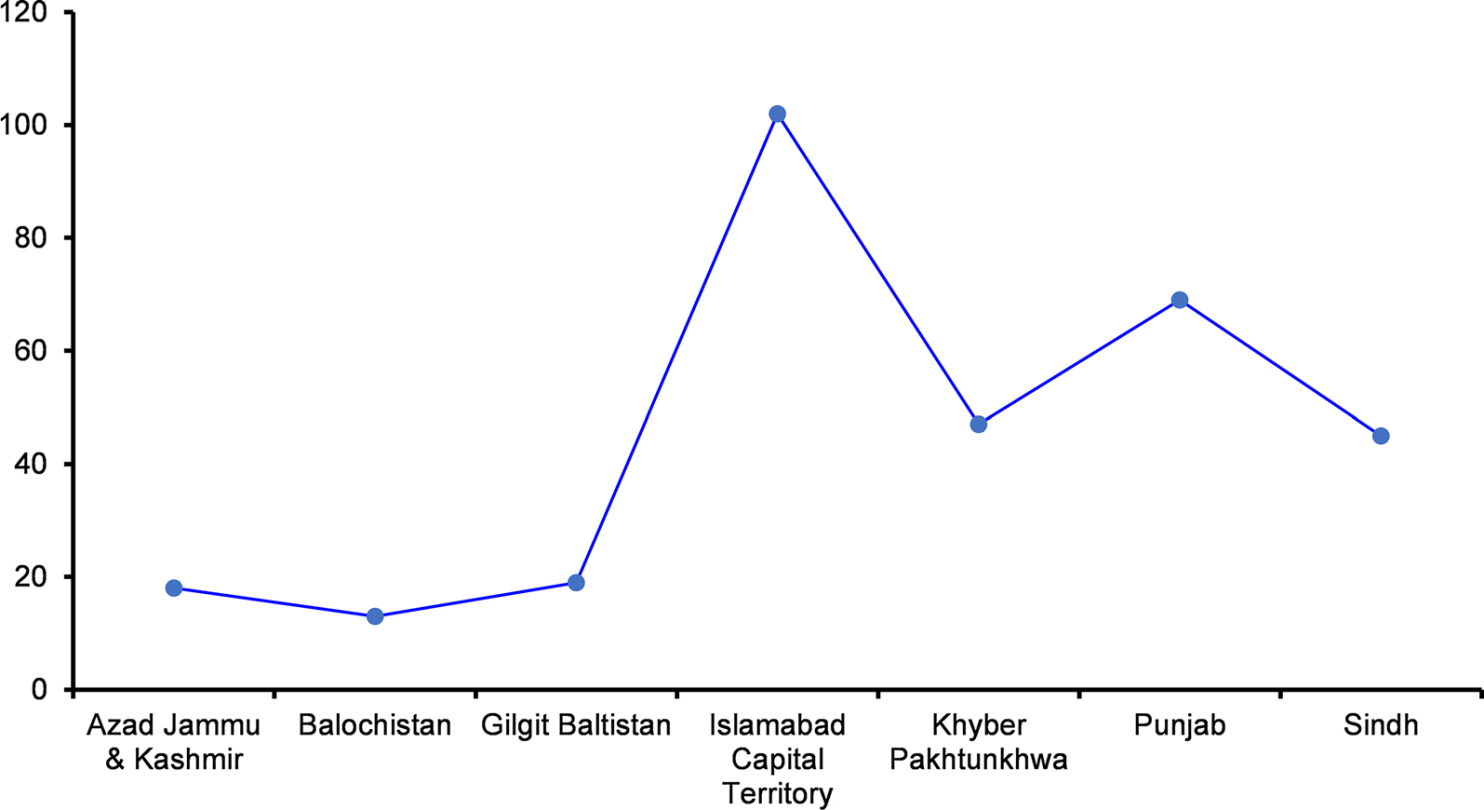
Distribution of participants’ responses by region (n = 313)

Figure 1 shows the distribution of participants’ responses by region.

Table 1 shows the demographic information of the participants. Out of 313 participants, 42.81% were male and 57.19% were female with a common age bracket of 20–40 years (83%). By designation most of the participants were general practitioners (34%).

**Table 1:** Demographic information of dental care professionals n = 313

Demographics	Numbers (%)
*Gender*	
Male	134 (43)
Female	179 (57)
*Age*	
20–30 years	181 (58)
31–40 years	77 (25)
41–50 years	34 (11)
50 years and above	21 (7)
*Qualification/designation*	
House officer	64 (20)
General practitioner	107 (34)
Postgraduate trainee	70 (22)
Consultant/specialist	72 (23)
*Which practice do you work in?*	
Government	97 (31)
Private hospital	113 (36)
Clinic	103 (33)

**Fig. 2:**
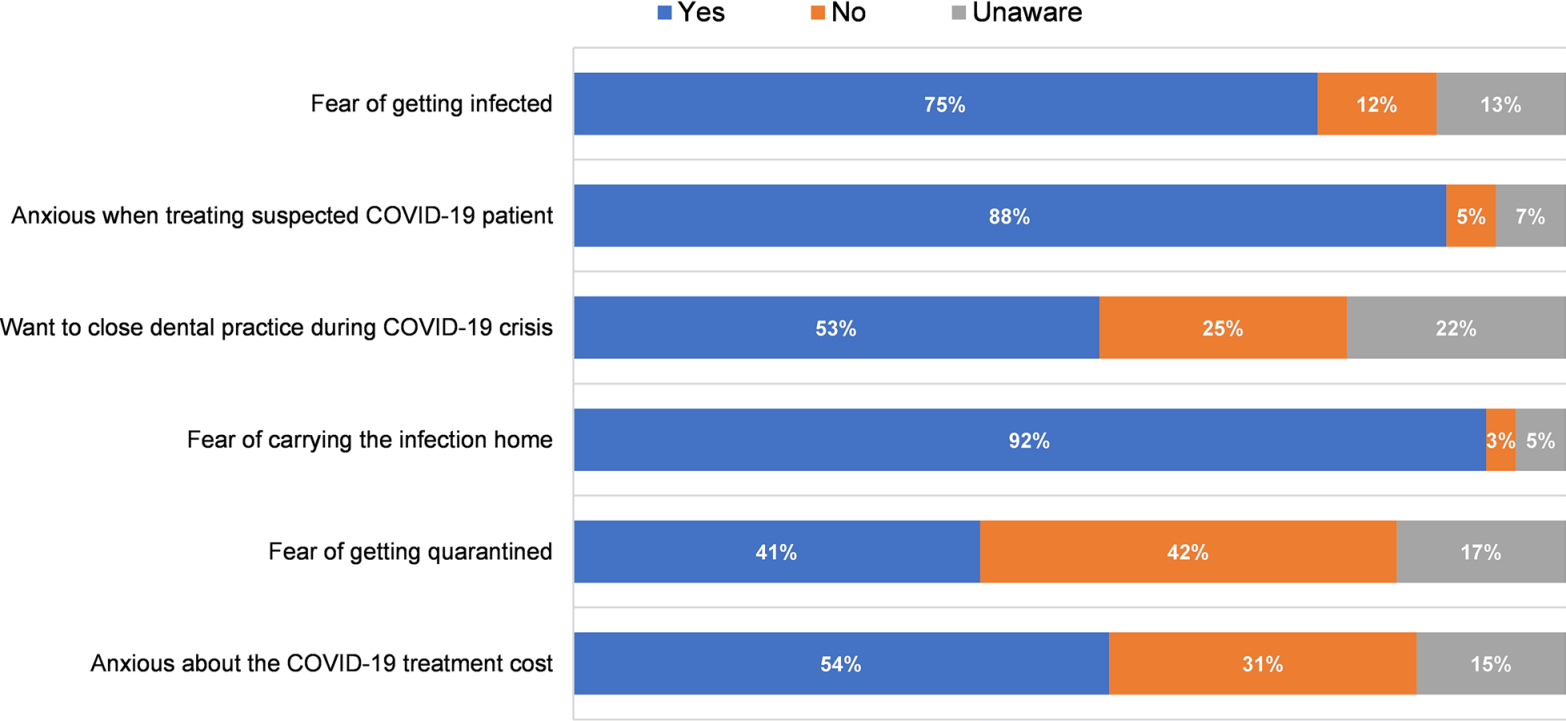
Fear and anxiety assessment of dental professionals

Figure 2 shows the level of fear and anxiety of dental professionals during COVID-19. Most of the dentists were afraid of getting infected with COVID-19 from a patient or a co-worker and were anxious while providing treatment to a suspected patient. Ninety-two percent had the fear that they would carry the infection back to their family.

**Fig. 3:**
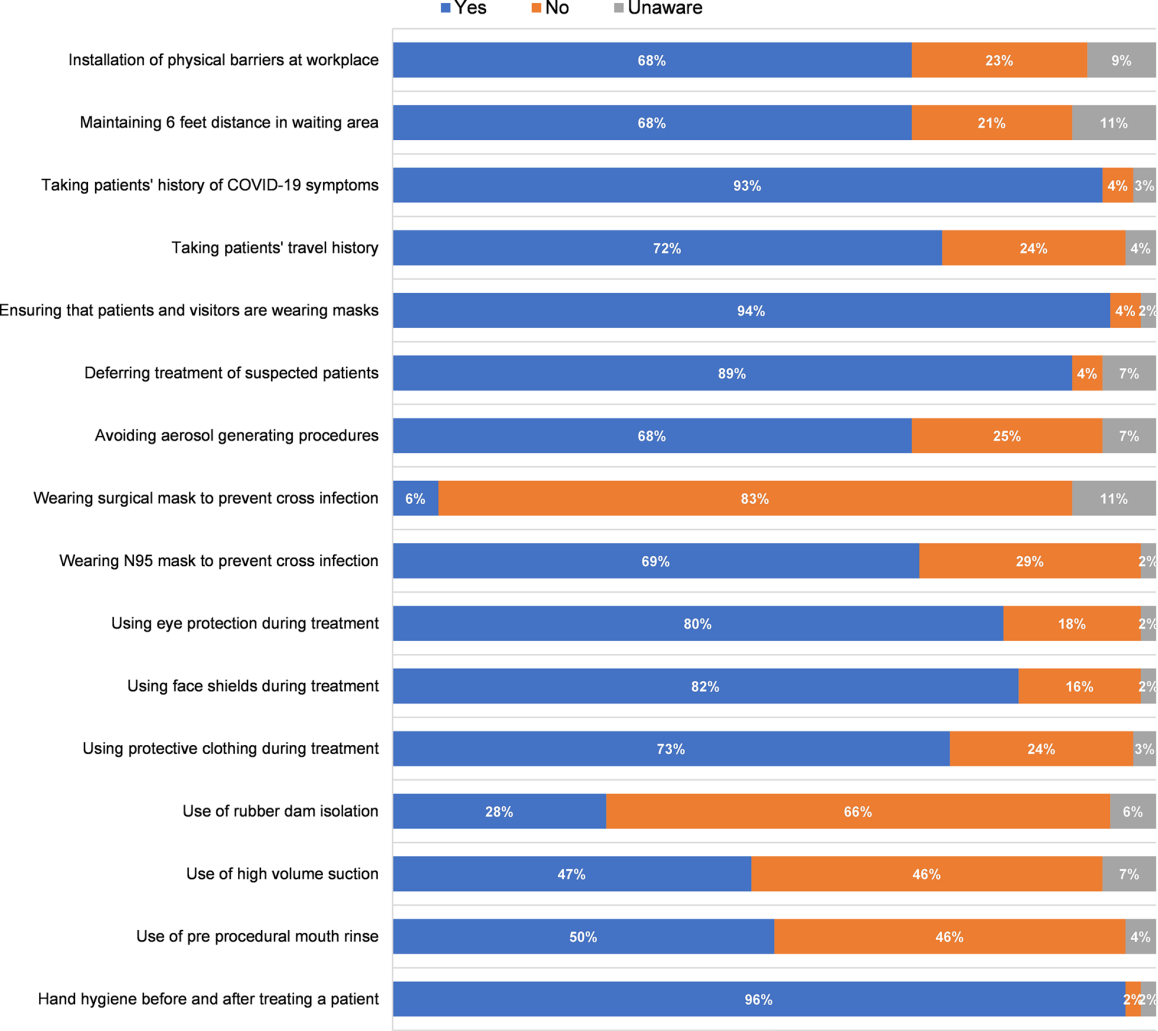
Knowledge and practice modification of Dentists during COVID-19

Figure 3 shows the knowledge and work practice modification of dental professionals about COVID-19. Majority of the of the dentists were taking patients’ history of fever, cough and body aches before performing dental treatment, 68% of the dentists were trying to avoid aerosol generating procedures whenever possible and prioritising minimally invasive dentistry. Most of the dentists were well aware of the CDC guidelines related to PPEs against the COVID-19 in their dental practice. Surprisingly, a very small number of dentists (28%) were using rubber dam isolation while performing aerosol generating procedures.

Regional variations were also noted in the level of awareness among the practicing dentists across Pakistan. In Balochistan, only 46.2% of the dentists were taking travel history, 38.5% were wearing N95 masks while treating a patient and 53.8% were using eye protection. None of the dentists (0%) were using rubber dam isolation, 15.4% were using high volume suction during dental treatment and asking the patient to rinse their mouth with an anti-bacterial mouth wash before treatment.

A large number of dentists (80.9%) in Khyber Pakhtunkhwa, wanted to close their dental practice until the number of COVID-19 cases start declining.

Association of awareness level with designation/qualification and workplace setting (private, government setup) was also evaluated. The correlation between the designation and awareness level was significant with a p value of 0.01, showing that the awareness level rises with rise in qualification. Similarly, a significant correlation was seen with work place setting and awareness level with a p value of 0.031, indicating that the doctors of private hospitals have more mean awareness score as compare to the ones working in government setup.

## Discussion

This is the first nationwide study that has been undertaken in Pakistan which looked into the fear and anxiety levels and also assessed dentists’ knowledge and practice modification (according to CDC, WHO, ADA guidelines) during the current pandemic. A survey conducted in 2012 showed that out of 13,000 dentists registered in Pakistan, only 8500 of them were actually working. However, the current study revealed that only 313 dentists across Pakistan participated and submitted the form. This low number depicts that most of the dentists were not working during the COVID-19 era, as the questionnaire was only to be filled by practicing dentists during that time. This could be due to the lockdown situation in the country or because the dentists were actually afraid to practice during that time.

In situations like the ongoing pandemic with increasing mortality rate, fear and anxiety are natural psychological implications [2]. The fear of getting infected while treating a patient or carrying the infection back home has been reported as a factor causing psychological trauma in healthcare workers [20, 21]. The current study revealed that a large number of dentists (75%) were afraid of getting infected in the workplace and even a larger number (92%) were afraid to carry the infection back home. Similar results were observed by a Turkish study which reported that 90% of the dentists were afraid of getting infected and 95% of them were concerned about carrying the virus to their family [1].

Taking patients history is very important specially during this ongoing pandemic since a COVID infected patient with acute symptoms is a potential source of infection. In the current study, 93% of the dentists in Pakistan were taking patients history of COVID-19 symptoms and 68% of them were avoiding aerosol generating procedures.

Dentists have always been at higher risk for airborne infections, therefore they should be even more cautious while attending patients during this current pandemic. Use of face shields and goggles is recommended to prevent spatter during a dental procedure. In Pakistan most of the dentists were well aware of the CDC guidelines regarding the use of PPEs (eyewear 80%, face shield 82% and protective clothing 73%). Surprisingly 69% of the dentists were using N95 masks while treating patients compared to only 12.36% Turkish dentists [1]. This high percentage may be because Pakistan being a third world country has received a lot of foreign aid during this time.

Dental dams have been recommended by CDC to minimise droplet spatter and aerosols especially during the COVID-19 pandemic. The current study revealed that only 28% of the dentists were using rubber dam isolation for aerosol generating procedures compared to 13.84% Turkish dentists [1]. This low percentage is because a lot of dentists find it difficult and time consuming using rubber dam on every patient. Regarding the use of rubber dam similar responses (14%) were seen in a survey conducted in 30 different countries [2].

According to CDC guidelines there is no published evidence regarding the clinical effectiveness of pre procedural mouth rinses (PPMRs) but they suggest its use to reduce the level of oral microorganisms in aerosols generated during dental procedure. Ahmed et al. reported 24% of the dentists were using pre procedural mouth rinse in different countries across the world [2], whereas in our study 50% of the Pakistani dentists were using mouth rinse before the procedure.

In the present study data was collected from different regions of Pakistan. Regional differences were also noted regarding the awareness in practice modification. In Balochistan lack of adherence to the CDC guidelines was noted as discussed in the results. This may be because of the distant nature and security situation in this region that the healthcare professionals are deprived of essential information and equipment [22].

One of the significant findings of our study was a positive correlation between awareness level and qualification/designation and workplace setting, showing that the awareness level rises with the rise in qualification/designation of the doctor. Similarly, the doctors of private hospitals have more mean awareness score as compare to the doctors of government setup. The major reason for this correlation is that private hospitals have always worked on maintaining their good repute by constituting and adhering to cross infection protocols and strict supervision, as they are adequately funded to achieve the patients trust and satisfaction.

One of the limitations of our study was that very few dentists were practicing during the pandemic and so a low response was obtained which can affect the generalizability of the study. Another limitation was that we could not get equal representation from all the regions of Pakistan. Only 13 dentists from Balochistan filled and submitted the questionnaire. Therefore, due to the limited data from this region, the results should be carefully interpreted.

## Conclusion

This study provides valuable data for developing strategies to overcome fear and spread awareness among Pakistani dentists, especially to the respective regions with poor compliance, to adhere to the current infection control guidelines. This will help contain the spread of disease and can eventually help lower the healthcare burden in a third world country like Pakistan.

## Supplementary Information

**Additional file 1:** Questionnaire used to assess the fear and compliance of Pakistani dentists with work practice modification during the COVID-19 pandemic.

## Data Availability

The datasets used and/or analysed during the current study are available from the corresponding author on reasonable request.

## Abbreviations

ADA: American Dental Association
CDC: Centers for Disease Control and Prevention
COVID-19: Coronavirus disease 2019
OPD: Out patient department
OSHA: Occupational Safety and Health Administration
PPE: Personal protective equipments
PPMR: Preprocedural mouth rinses
SPSS: Statistical Package for the Social Sciences
UNDP: United Nations Development Programme
WHO: World Health Organization
