# Supplementary material for: Impact of COVID-19 on Pakistani dentists: a nationwide cross sectional study
Source: BMC Oral Health. 2021 Feb 10;21:59. doi: 10.1186/s12903-021-01413-6 (PMC7874993; doi:10.1186/s12903-021-01413-6)
Supplement: Supplementary file 1 — Additional file 1: Questionnaire used to assess the fear and compliance of Pakistani dentists with work practice modification during the COVID-19 pandemic. [file 12903_2021_1413_MOESM1_ESM.docx]

**IMPACT OF COVID-19 ON PAKISTANI DENTISTS: A NATIONWIDE CROSS SECTIONAL STUDY**

1. **Age**
   1. 20-30 years
   2. 31-40 years
   3. 41-50 years
   4. 50 years and above
2. **Gender**
   1. Male
   2. Female
3. **In which region are you practicing?**
   1. Islamabad Capital Territory
   2. Punjab
   3. Sindh
   4. Balochistan
   5. Khyber Pakhtunkhwa
   6. Azad Jammu & Kashmir
   7. Gilgit Baltistan
4. **Which practice do you work in?**
   1. Government
   2. Private hospital
   3. Clinic
5. **For how many years are you practicing dentistry?**
   1. Less than 5 years
   2. 5 to 10 years
   3. 10 to 20 years
   4. 20 years and above
6. **Qualification**
   1. House Officer
   2. General practitioner
   3. Postgraduate Trainee
   4. Consultant/Specialist
7. **Are you afraid of getting infected with COVID-19 from a patient and co-worker?**
   1. Yes
   2. No
   3. Neutral
8. **Are you anxious when providing treatment to a patient who is coughing or suspected of being infected with COVID-19?**
   1. Yes
   2. No
   3. Neutral
9. **Do you want to close your dental practice until the number of COVID-19 cases starts declining?**
   1. Yes
   2. No
   3. Neutral
10. **Do you feel nervous when talking to patients in close vicinity?**
    1. Yes
    2. No
    3. Neutral
11. **Do you have a fear that you could carry the infection from your dental practice back to your family?**
    1. Yes
    2. No
    3. Neutral
12. **Are you afraid of getting quarantined if get infected?**
    1. Yes
    2. No
    3. Neutral
13. **Are you anxious about the cost of treatment if you get infected?**
    1. Yes
    2. No
    3. Neutral
14. **Do you feel afraid when you hear that people are dying because of COVID-19?**
    1. Yes
    2. No
    3. Neutral
15. **Have you installed physical barriers at reception areas to limit close contact between the personnel and potentially infectious patient?**
    1. Yes
    2. No
    3. Don’t know
16. **Are you limiting inter patient transmission by placing chairs in the waiting area at least six feet apart?**
    1. Yes
    2. No
    3. Don’t know
17. **Are you taking patient’s history of fever, cough and body aches before performing dental treatment?**
    1. Yes
    2. No
    3. Don’t know
18. **Are you currently asking every patient’s travel history before performing dental treatment?**
    1. Yes
    2. No
    3. Don’t know
19. **In the current pandemic are you ensuring that the patient and visitors are wearing face masks?**
    1. Yes
    2. No
    3. Don’t know
20. **Are you deferring dental treatment of patients showing suspicious symptoms?**
    1. Yes
    2. No
    3. Don’t know
21. **Are you trying to avoid aerosol generating procedures whenever possible in your dental practice and prioritising minimally invasive/atraumatic restorative techniques (hand instruments only) ?**
    1. Yes
    2. No
    3. Don’t know
22. **Do you think surgical mask is enough to prevent cross infection of COVID-19?**
    1. Yes
    2. No
    3. Don’t know
23. **Do you think N95 masks should be routinely worn in dental practice due to the current outbreak?**
    1. Yes
    2. No
    3. Don’t know
24. **Have you ever worn an N95 mask while treating a patient in your dental practice?**
    1. Yes
    2. No
    3. Don’t know
25. **Are you currently using eye protection while treating patients?**
    1. Yes
    2. No
    3. Don’t know
26. **Are you currently using face shields while treating patients?**
    1. Yes
    2. No
    3. Don’t know
27. **Are you currently wearing protective clothing while treating patients?**
    1. Yes
    2. No
    3. Don’t know
28. **Do you use rubber dam isolation for every patient?**
    1. Yes
    2. No
    3. Don’t know
29. **Do you use high volume suction in your practice for every patient?**
    1. Yes
    2. No
    3. Don’t know
30. **Do you ask every patient to rinse his/her mouth with antibacterial mouthwash before treatment?**
    1. Yes
    2. No
    3. Don’t know
31. **Do you wash hands with soap and water/sanitiser before and after treatment of every patient?**
    1. Yes
    2. No
    3. Don’t know
32. **Are you aware of which authority to contact if you come across a patient with suspected COVID-19 infection?**
    1. Yes
    2. No
    3. Don’t know
